# Progressive Multifocal Leukoencephalopathy in a Chemotherapy-Naive Patient With Chronic Lymphocytic Leukemia: A Case Report

**DOI:** 10.7759/cureus.32912

**Published:** 2022-12-25

**Authors:** Nina Jancar, Filipa Sousa Gonçalves, José Duro, Mariana Lessa Simões, Patrício Aguiar

**Affiliations:** 1 Internal Medicine, Hospital de Santa Maria, North Lisbon University Hospital Center (CHULN), Lisbon, PRT

**Keywords:** severe neurologic impairment, white matter lesions, hiv-negative progressive multifocal leukoencephalopathy, chronic lymphocytic leukemia (cll), jc virus

## Abstract

Progressive multifocal leukoencephalopathy is a rare, progressive demyelinating disease of the central nervous system caused by reactivation and replication of the John Cunningham (JC) virus in cerebral oligodendrocytes. The JC virus is a small ubiquitous polyomavirus that can be detected in up to 50% of the adult population. It affects almost exclusively immunocompromised patients and is generally observed in patients with acquired immunodeficiency syndrome and patients with hematologic malignancies and autoimmune or chronic inflammatory diseases medicated with immunosuppressive and immunomodulatory drugs. However, it is rarely described in patients with hematologic malignancies, not undergoing chemotherapy or immunosuppressive therapy. It has a poor prognosis, and the treatment is based on restoring the immune system, given that no specific antiviral treatment is available. We present a case of a chemotherapy-naive patient with chronic lymphocytic leukemia associated with progressive multifocal leukoencephalopathy.

## Introduction

Progressive multifocal leukoencephalopathy (PML) is a rare, typically rapidly progressive disease of the central nervous system caused by a ubiquitous, small polyomavirus, the John Cunningham virus, which occurs almost exclusively in immunocompromised patients [1-5]. Epidemiologic studies are scarce; according to some reports, its incidence in the general population is 0.12/100000, but it is higher in risk groups and depends on the underlying condition [6,7]. It is most frequently observed in patients with acquired immunodeficiency syndrome (AIDS). Still, it can also affect patients with hematologic and solid malignancies, autoimmune diseases, primary immunodeficiencies, solid organ, and hematopoietic stem cell transplant patients, as well as patients undergoing immunomodulatory and immunosuppressive treatments such as rituximab, natalizumab, efalizumab, infliximab, etc. [2,6]. The clinical manifestations are heterogeneous, given the multifocal demyelinating lesions, and can vary from motor symptoms and seizures to cognitive decline [2,7]. The diagnosis is based on clinical findings, typical neuroradiological findings, the presence of the JC virus in cerebrospinal fluid, or characteristic histopathologic findings on a biopsy of the central nervous system (CNS) [7]. The treatment is based on the immune system's reconstitution, as no specific antiviral therapy is available [8]. Treatment with checkpoint inhibitors has been applied recently [9] but has shown mixed outcomes in patients with hematologic malignancies [10].

## Case presentation

**Figure 1:**
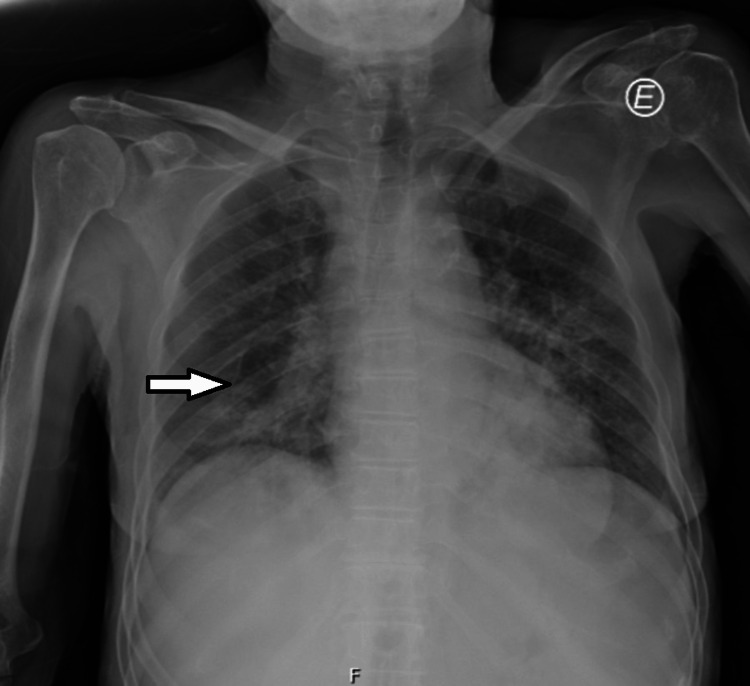
Chest radiograph at admission, showing a diffuse, bilateral interstitial infiltrates, and consolidation in the lower third of the right hemithorax

We present a case of a 77-year-old female patient who did not report any relevant medical history or take any previous medication. She presented with generalized discomfort, exertional dyspnea, fatigue, anorexia, and weight loss (about 16% of the total body weight), which started insidiously in the four months before hospitalization. Additionally, she reported diminished visual acuity, left upper limb paresthesia, muscle weakness, vespertine fever, and night sweats, which started one month before hospitalization. Lymphocytosis (28000/uL) was documented in a laboratory evaluation about a month before the hospitalization. An abdominal mass and splenomegaly were palpable on physical examination, and left hemiparesis (grade III) with a positive ipsilateral Babinski sign, hyporeflexia of the lower left member, left-sided visual neglect, and left-sided hypoesthesia were observed. Laboratory tests revealed normocytic anemia (10.5g/dL), leucocytosis (19370/uL) with absolute lymphocytosis (18000/uL, of which 7740/uL were B lymphocytes), the dysmorphic lymphoid population on peripheral blood smear and hypoxemia on blood gas analysis (63mmHg). A chest radiograph showed a bilateral, diffuse interstitial infiltrate and consolidation in the lower third of the right hemithorax. A cranial computerized tomography (CT) was also performed. It revealed extensive areas of vasogenic edema in the periventricular white substance adjacent to the lateral ventricles, corpus callosum, and subcortical substance of the superior right frontal circumvolution. Figure 1 shows chest radiograph at admission.

**Figure 2:**
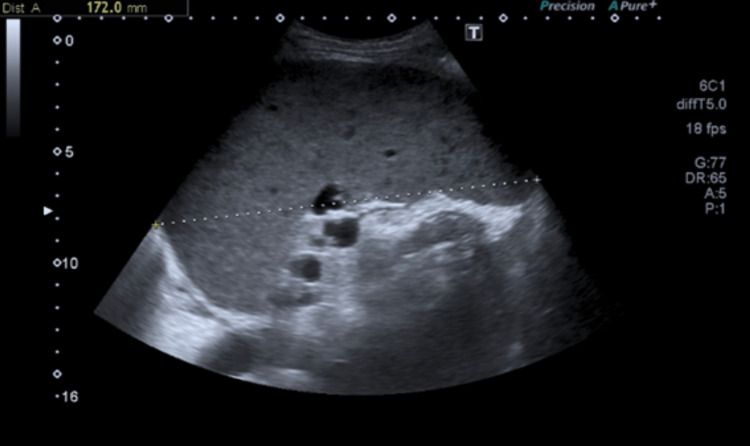
Abdominal ultrasound showing splenomegaly (splenic length of 17.2cm) with nodular images Cm: Centimeter

The patient was started on amoxicillin/clavulanic acid and azithromycin for community-acquired pneumonia, and further diagnostic tests were performed. Peripheral blood immunophenotype showed a monoclonal B lymphocyte population and was suggestive of mature B cell neoplasia/chronic lymphocyte leukemia; protein electrophoresis showed hypogammaglobulinemia, and abdominal ultrasound (Figure 2) revealed moderate splenomegaly with nodular images.

**Figure 3:**
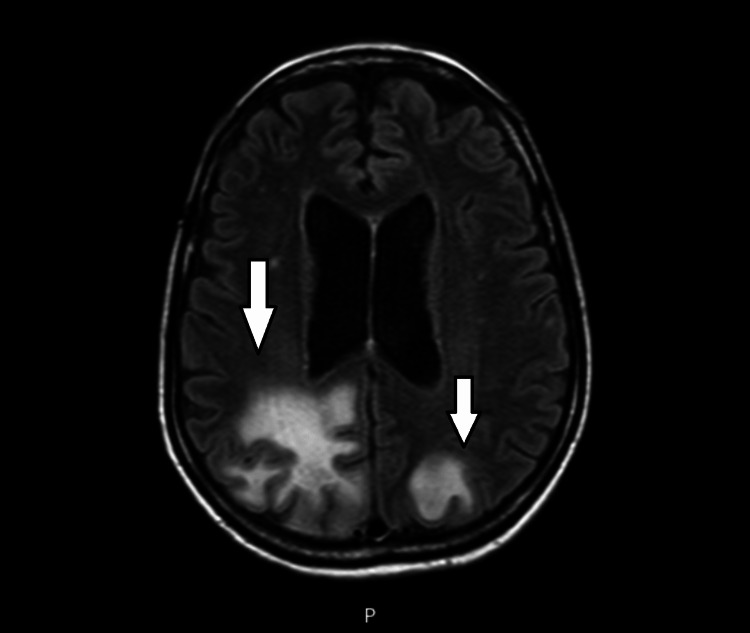
Cranial magnetic resonance imaging (MRI) showing biparietal intra-axial white substance lesions, hyperintense in T2

The diagnosis of chronic lymphocytic leukemia Rai 3 Binet A was confirmed. Given the neurological deficits, a cranial magnetic resonance was performed and showed biparietal intra-axial white substance lesions that were hyperintense in T2 (Figure 3) and hypointense in T1. The lesions were more pronounced in the right hemisphere, extending to the corpus callosum and the mesencephalon, with a discrete mass effect. 

A lumbar puncture was performed, and it showed the presence of lymphocytes and a discrete elevation of IgG in the cerebrospinal fluid (CSF). A CSF bacterial culture, ADA, AFP, NSE, *Cryptococcus,* and *Brucella *serologies were negative. The cytological examination, CSF immunophenotype, and tests for mycobacteria, herpes virus, syphilis, neurotropic viruses, and Lyme disease were all unremarkable. Hepatitis B, C, and human immunodeficiency virus (HIV) serologies were all negative. There was evidence of past infection by herpes simplex virus (HSV) 1, cytomegalovirus (CMV), and toxoplasmosis.

A JC virus polymerase chain reaction (PCR) test was performed in the cerebrospinal fluid and was positive, which confirmed the diagnosis of PML. The patient was started on immunoglobulin and mirtazapine 15 mg per day. Given the rapid progress of the neurologic symptoms and the worsening performance status, the patient did not start any specific therapy for leukemia and was transferred to a palliative medicine unit, where she died approximately two weeks after the diagnosis of PML.

## Discussion

Progressive multifocal leukoencephalopathy is a rare, rapidly progressive disease of the central nervous system caused by a ubiquitous, small polyomavirus, JC virus, first described in 1958 in a series of patients with untreated chronic lymphocytic leukemia (CLL) and Hodgkin’s disease [2,7]. It occurs almost exclusively in immunocompromised patients, most commonly patients with hematologic or solid malignancies, patients with acquired or primary immunodeficiency syndromes, and patients who have undergone an organ transplant or are undergoing immunosuppressive or immunomodulatory therapy [1-5].

The initial asymptomatic infection usually occurs in childhood [2]. It is followed by a latent/persistent infection of the kidneys, as well as hematopoietic organs, where it undergoes reactivation and transformation into a neurotropic virus, enabling the invasion of the nervous system and replication in the glial cells, ultimately causing multifocal demyelination [2,7-8]. Given the multifocal involvement, the classical clinical presentation is characterized by progressively worsening multifocal neurologic symptoms, which depend on the localization of the lesions and can present with motor and sensory deficits, ataxia, aphasia, visual changes, seizures, as well as cognitive and behavioral abnormalities, headache, vertigo, and parkinsonism [8,11]. 

Our patient presented with typical B symptoms in the months before the hospitalization, and splenomegaly, anemia, and absolute lymphocytosis, with an immunophenotype suggestive of CLL-B, were documented at admission, which was compatible with the diagnosis of chronic lymphocytic leukemia stage III A (classification of Rai Binet). Additionally, she referred multifocal neurologic symptoms, which she had noted about a month before the hospitalization, ultimately motivating her to seek medical attention. Rapid progression of the symptoms was documented during the hospitalization. Despite the inherent secondary immunosuppression in patients with CLL due to impaired humoral and cellular immunity [12], the significant risk factor for PML in hematologic patients is immunosuppressive chemotherapy, radiotherapy, or hematopoietic stem cell transplant [7,13]. Nowadays, the disease is rarely described in patients not undergoing therapy [8]. 

Prompt diagnosis is essential, given the rapid progression of the disease, and is based on clinical, radiological, and microbiological evidence or documentation of typical histopathologic characteristics from a central nervous system biopsy. Magnetic resonance imaging is the best imaging method. It usually shows multifocal, subcortical white matter lesions in the frontal, but more commonly in the temporal and occipital lobes, typically hyperintense on T2 weighted fluid-attenuated inversion recovery (FLAIR) sequences and hypointense lesions on T1 weighted images. However, its radiological appearance can be heterogeneous and depends on the underlying etiology [2,11]. Cerebrospinal fluid analysis and documentation of the virus are essential for the diagnosis, although a negative PCR for the JC virus in the cerebrospinal fluid does not exclude the diagnosis [8]. Our patient presented with a typical neuroradiological pattern, with biparietal intra-axial white substance lesions, hyperintense in T2 and hypointense in T1, more pronounced in the right hemisphere, with extension to the corpus callosum and the mesencephalon, and a discrete mass effect. Other diagnoses, such as involvement of the central nervous system in the setting of hematologic disease, and viral, bacterial, and opportunistic CNS infections, were considered differential diagnoses and were all excluded. Given the symptoms, the typical radiological findings, and the confirmation of the JC virus in the cerebrospinal fluid, the diagnosis of multifocal progressive encephalopathy was confirmed.

The treatment of PML is based on immune reconstitution, as no specific antiviral therapy is available [6]. Although the survival rate of PML has improved drastically, especially in HIV patients, the mortality rate continues to be elevated when a rapid reversal of immune deficiency is not possible, which occurs in patients with hematologic malignancies [6,8,10]. Immune checkpoint inhibitors that target programmed cell death pathways, such as pembrolizumab and nivolumab, are promising treatment options as they upregulate T-cell activation and cell-mediated immune response [8-10]. Moreover, they seem to reduce the JC viral load, leading to disease stabilization and symptom improvement [14]. We opted to start the patient on immunoglobulin, given the hypogammaglobulinemia she presented, and mirtazapine, a 5HT2A receptor antagonist. According to in vitro studies, the infection of glial cells by the JC virus is mediated by the 5HT-2A receptors, and its antagonist might impede its entry into CNS cells and, thus, it's spread [15]. Given the rapid progress of our patient’s symptoms, poor performance status, and poor prognosis, it was decided that no specific treatment for leukemia or PML would be started after a hematology and neurology consultation since the risk and adverse effects of the therapy did not outweigh the benefit. The patient passed away about two months after the beginning of the neurologic symptoms and about two weeks after the diagnosis of PML.

## Conclusions

Progressive multifocal leukoencephalopathy is a rare, rapidly progressing, and often fatal demyelinating disease seen almost exclusively in immunocompromised patients. It is rarely described in patients with hematologic malignancies who underwent no treatment. It should be suspected and ruled out in patients with a new onset of neurologic symptoms, given the prognostic impact of early recognition. Immune reconstitution is paramount in treating the disease and, when not possible, is associated with a poor prognosis, as seen in our case report.
